# TOSSIT: A low-cost, hand deployable, rope-less and acoustically silent mooring for underwater passive acoustic monitoring

**DOI:** 10.1016/j.ohx.2022.e00304

**Published:** 2022-04-15

**Authors:** Daniel P. Zitterbart, Alessandro Bocconcelli, Miles Ochs, Julien Bonnel

**Affiliations:** Applied Ocean Physics and Engineering Department, Woods Hole Oceanographic Institution, Woods Hole, MA, USA

**Keywords:** Ocean ambient noise, Mooring systems, Soundscape, Underwater sound, Bioacoustics, Oceanography, Acoustical Oceanography

## Abstract

Passive Acoustic Monitoring (PAM) has been used to study the ocean for decades across several fields to answer biological, geological and meteorological questions such as marine mammal presence, measures of anthropogenic noise in the ocean, and monitoring and prediction of underwater earthquakes and tsunamis.

While in previous decades the high cost of acoustic instruments limited its use, miniaturization and microprocessor advances dramatically reduced the cost for passive acoustic monitoring instruments making PAM available for a broad scientific community. Such low-cost devices are often deployed by divers or on mooring lines with a surface buoy, which limit their use to diving depth and coastal regions.

Here, we present a low-cost, low self-noise and hand-deployable PAM mooring design, called TOSSIT. It can be used in water as deep as 500 m, and can be deployed and recovered by hand by a single operator (more comfortably with two) in a small boat. The TOSSIT modular mooring system consists of a light and strong non-metallic frame that can fit a variety of sensors including PAM instruments, acoustic releases, additional power packages, environmental parameter sensors. The TOSSIT’s design is rope-less, which removes any risk of entanglement and keeps the self-noise very low.

## Graphical abstract

**Figure:**
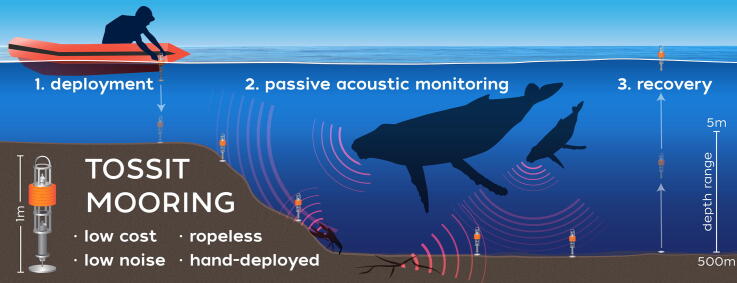

## Hardware in context

Passive Acoustic Monitoring (PAM) in the ocean has become a standard technique across the oceanographic community. On top of historical military applications, PAM is now widely used for biological, geological and meteorological questions such marine mammal occurrence and population density estimation [1], ocean ambient noise characterization [2], [3], soundscape measurements on coral reef to assess biological activities [4], marine biodiversity assessment [5], seismic monitoring [6], acoustical meteorology [7], estimation of water column or seafloor geoacoustic properties [8], [9].

A current trend in engineering is to (try to) replace large, expensive and highly sophisticated systems by swarms of smaller, cheaper, but more basic systems. During recent years, the marine research community has started to follow this trend for various applications, e.g. [10]. Of particular interest here is that a plethora of different low-cost (<$5000USD) PAM recorders have been developed (Ocean Instruments SoundTrap, DesertStar microMARS, Develogic SVmini, RTsys Porpoise, Open Acoustic Devices Hydromoth) and made PAM available across a much larger scientific community, including smaller organizations and NGO’s. These low-cost recorders are usually limited to water depths less than 500 m and have an endurance of a few days to a few months. Hence, installations often are conducted from small vessels and deployed by divers or on mooring lines with surface expressions, and not within highly elaborate mooring deployments. This limits the use of such devices to diving depth or, if simple mooring lines with surface expression are used, reduces data quality due to mooring induced noise, while low self-noise installments are key for successful PAM data collection.

Fixed PAM recorders can be deployed in four fashions [11]:

-as bottom mounted recorders without surface expression, which require a fixed weighted frame on the bottom of the ocean with an acoustic release and back-up floatation;

-as bottom mounted recorders tethered to a surface buoy for recovery;

-as bottom mounted recorders tethered to an anchor without release, deployed and recovered by diver;

-as on-line sensors on a traditional surface mooring connected to the mooring line and placed at a pre-determined depth in the water column.

Mooring with line always bear the risk of strumming [12], [13], vibration of the mooring cable caused by currents. The noise introduced by strumming negatively impacts the acoustic data quality. This is usually mitigated by the used of faired cable, which comes at high cost. The use of a surface expression, especially in high-current coastal areas bears the problem that wave induced motion and pull on the surface mooring causes significant noise, making it unsuitable for PAM noise studies. This can be mitigated using stretch hoses but come at high cost as well. Bottom mounted recorders with or without surface mooring currently have to be in depth that can be reached by divers to ensure successful recovery or be attached to an acoustic release. In high-turbidity (low-visibility) areas of the coast, a surface expression is a must if the mooring is required in order to be located and recovered by a diver. Small surface expressions in coastal areas are often subject to negative interactions with commercial fishing gear and theft and it is favorable to avoid these. In areas where entanglement of baleen whales is likely, rope-less mooring systems are favorable for conservation reasons.

Here, we present the TOSSIT (“toss it”) mooring assembly. TOSSIT is a low-cost, low-noise PAM mooring assembly, designed to be deployed by hand in water depths of up to 500 m. We publish the design files in hope that they will be useful to the low-cost underwater passive acoustic monitoring community.

The TOSSIT concept is of interest for all the marine applications that have historically relied on bottom-moored single-hydrophone measurements, notably bioacoustics [1], [2], [5], [14] or soundscape studies [2], [3], [4], [7], [15]. Classically, most other ocean acoustics experiments involve the use of synchronized arrays of sensors to perform spatial and temporal filtering. However, recent advances in data science and signal processing now enables localizing a source or characterizing the propagation environment with a single hydrophone [16], [17], [18], [19], [20], [21]. Those applications would also greatly benefit from the TOSSIT concept. In particular, TOSSIT's low-cost makes it possible to use a sparse and distributed array of dozens of (unsynchronized) TOSSITs to fully cover an experimental area of hundreds of km^2^. This could change the way we design ocean acoustic experiments for both marine mammal monitoring and environmental inversion (see section 7 for practical examples).

## Hardware description

The mooring system was designed to be deployed by hand from small boats for passive acoustic monitoring applications. It consists of the mooring frame, built-in buoyancy, a commercial of the shelf (COTS) passive acoustic recorder (Soundtrap ST300) and a COTS underwater acoustic release (Vemco Ascent AR). The passive acoustic recorder can be easily exchanged by any other model up to a maximum diameter of 125 mm and adjustment of one piece. For longer acoustic recorders such as a Soundtrap 600 four standoffs would need to be enlongated as well.

The TOSSIT mooring is specifically adapted to the Vemco Ascent AR acoustic release which we modified to remove movement to minimize self noise. Use of the Vemco Ascent AR acoustic release requires a deck unit to remote control the release function. We do not consider the deck box part of the TOSSIT mooring, and its therefore not included into the hardware description.

A key aspect of the TOSSIT mooring is that it is entirely rope-less, and has no moving parts, which both are crucial to reduce self-noise, hence increase the data quality. Rope-less design minimizes the self-noise by avoiding rope-line strumming and enables to use higher sensitivity settings and removes the risk of entanglement for marine mammals in the mooring lines.

The modular design and low-weight of the TOSSIT mooring allow it to be transported in checked luggage and it can be assembled with a single 3/16'' Allen Key and an adjustable wrench. Depending on how the anchor is attached, another large adjustable wrench or right sized nut driver are necessary.

We foresee that TOSSIT moorings can be useful for following scenarios:

• Low-cost passive acoustic monitoring for smaller organizations that want to conduct acoustic studies in water deeper than what is feasible for diver deployments.
• Low-cost PAM deployment in areas of high entanglement risk (e.g. Manatees, North Atlantic right whales)
• Large-scale deployment of TOSSIT moorings to create a passive acoustic monitoring distributed array that can be used for localization and environmental estimation.

**Fig. 1:**
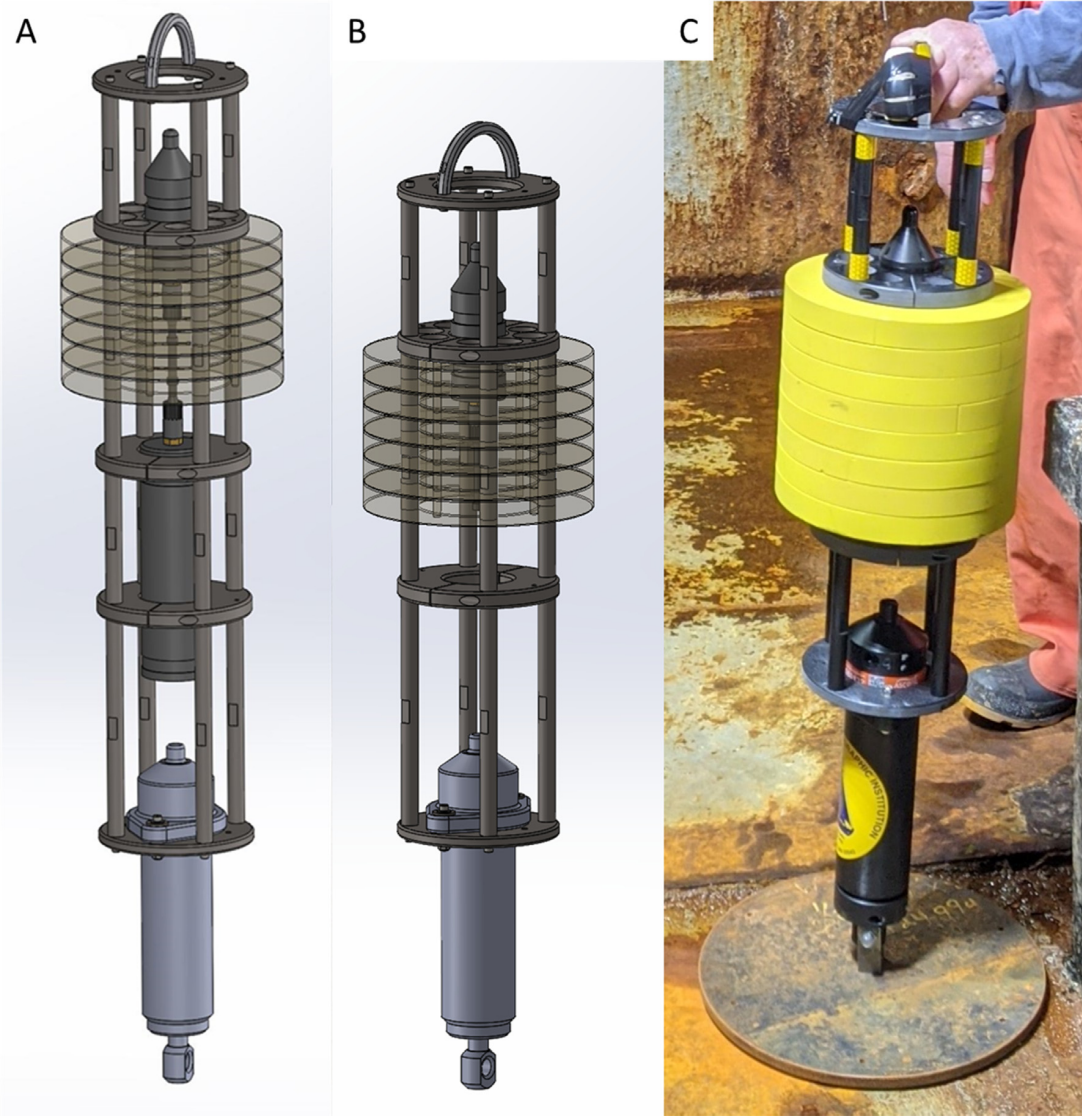
A) Drawing of the assembled TOSSIT mooring in a four section configuration with optional battery pack. B) Drawing of the assembled TOSSIT mooring in a three section configuration without optional battery pack. C) Picture of a TOSSIT mooring in a three section configuration and anchor weight attached.

The TOSSIT mooring frame is of modular design and reuses the same parts for ease of production, assembly and spare parts. It consists of a series of round plates which are attached to each other by Delrin rods (Fig. 1). Its modular design allow TOSSIT to be used with either three or four sections. In a three sections configuration the frame holds the acoustic release and the acoustic recorder (Fig. 1B), while in a four sections configuration, it additionally holds a battery pack (Fig. 1A) for increased duration of the acoustic recorder. The top plate holds the handle, which can be used to lower and recover the TOSSIT. The second plate holds the Soundtrap. The build in buoyancy (buoyancy foam plates) is slipped on the posts between plates two and three. Plates three (and optionally four) are used for stability and/or to secure the battery pack, while the lowermost plate hold the acoustic release.

The entire system is designed for operations in up to 500 m, which currently is limited by the depth rating of the acoustic release (500 m) and the acoustic recorder (500 m). The selected buoyancy foam is rated to 900 m. When using a TOSSIT mooring in depths less than 60 m, a line-pack can be included to allow for recovery of the weight. The line-pack works like a pop-up rope canister. Up to 100 m of line can be spooled on the line-pack, and once the acoustic release is surfacing, the line unspools itself during ascent. This line can then be used to retrieve the anchor weight. Line-packs to deeper depths have proved impractical and when using a TOSSIT in deeper depth, we suggest to simply leave the anchor weight at the seafloor.

TOSSIT was designed to be handled by hand, hence the weight and buoyancy are kept to a minimum. A typical TOSSIT mooring anchor weighs ∼ 12–15 kg. Any weight can be used as long as it provides a rigid eye-like attachment where the acoustic release lug can be attached to (Fig. 1C). If several TOSSITs are deployed, we advise for plate-like weights (see Fig. 1C) since those can easily be stacked, thus minimizing the required deck space.

## Design files

The design file lists for the hardware are shown in Table 1. All design files can be accessed through Zenodo https://10.5281/zenodo.5632099. Table 2..

**Table 1:** Hardware design files.

Design file name	File type	Open source license	Location of the file
*100,438 TOSSIT HYDROPHONE MOORING*	*CAD & PDF*	*OHL*	*https://doi.org/10.5281/zenodo.5632099*
*100,440 FLOTATION RING*	*CAD & PDF*	*OHL*	*https://doi.org/10.5281/zenodo.5632099*
100,441 LONG STANDOFF	*CAD & PDF*	*OHL*	*https://doi.org/10.5281/zenodo.5632099*
100,443 END PLATE	*CAD & PDF*	*OHL*	*https://doi.org/10.5281/zenodo.5632099*
100,444 VEMCO MOUNT BUSHING	*CAD & PDF*	*OHL*	*https://doi.org/10.5281/zenodo.5632099*
100,451 BATTERY HOUSING CLAMP	*CAD & PDF*	*OHL*	*https://doi.org/10.5281/zenodo.5632099*
100,452 SHORT STANDOFF	*CAD & PDF*	*OHL*	*https://doi.org/10.5281/zenodo.5632099*
100,453 ST300 HOUSING CLAMP	*CAD & PDF*	*OHL*	*https://doi.org/10.5281/zenodo.5632099*
100,500 HANDLE	*CAD & PDF*	*OHL*	*https://doi.org/10.5281/zenodo.5632099*
100,532 RELEASE LINK COLLAR	*CAD & PDF*	*OHL*	*https://doi.org/10.5281/zenodo.5632099*
100,533 ANTI ROTATION COLLAR	*CAD*	*OHL*	*https://doi.org/10.5281/zenodo.5632099*
100,601 TOSSIT LINE PACK	*CAD*	*OHL*	*https://doi.org/10.5281/zenodo.5632099*
100,602 TOSSIT ANTI BACKLASH COLLAR	*CAD & PDF*	*OHL*	*https://doi.org/10.5281/zenodo.5632099*

**Table 2:** Bill of Materials.

Part Number Designator	Part Name	Quantity Needed Per Assemb	,ost Per Unit - USE	Total Cost - USD	Source	Material Type
SOUNDTRAP ST300	Soundtrap ST300 STD	1	3100	3100	Ocean Instruments NZ	COTS Ectmnics
VEMCO ASCENT AR	/emco AscentAcoustic Release	1	5000	5000	Oceans Research	COTS Ectronics
100,440	FLOTATION RING	8	50	400	Stock DLB International AB, Finsihed part to be cut from stock using waterjet machine	Divinycell PVC Closed Cell Foam
100,441	LONG STANDOFF	8	25	200	Machine Shop	Delrin (Acetal)
100,443	END PLATE	2	25	50	Machine Shop	Delrin (Metal)
100,444	VEMCO MOUNT BUSHING	2	25	50	Machine Shop	Delrin (Metal)
100,451	BATERYHOUSINGCLAMP	4	50	200	MachineShop	Delrin(Acetal)
100,452	SHORT STANDOFF	8	25	200	Machine Shop	Delrin (Metal)
100,453	5 T300 HOUSING CLAMP	2	50	100	Machine Shop	Delrin (Metal)
100,500	HANDLE	1	50	50	MachineShop	6061-T6Juminum
100,532	RELEASE LINK COLLAR	1	40	40	McMaster Carr + Machine Shop	Delrin (Metal)
100,533	ANTIROTATIONCOLLAR	1	100	100	MachineShop	Delrin(Metal)
100,594	LINE PACK RE WINDER	1	500	500	Machine Shop	Delrin (Metal)
100,601	TOSSITLINEPACK	1	500	500	MachineShop	Delrin(Metal)
92185A517	Socket Head Cap Screw 114– 20 Thread Size, 2 Long	2	1.19	2.38	McMaster Carr or other Hardware Vendor	3l6StainlessSteel
92185A544	Socket Head Cap Screw 114– 20 Thread Size, 1–114 Long	12	0.375	4.5	McMaster Carr or other Hardware Vendor	316 Stainless Steel
92185A540	Socket Head Cap Screw 114– 20 Thread Size, 314 Long	2	0.306	0.612	McMaster Carr or other Hardware Vendor	316 Stainless Steel
92186A569	Socket Head Cap Screw 114– 20 Thread Size,8 Long	4	4.86	19.44	McMaster Carr or other Hardware Vendor	316 Stainless Steel
90575A546	Threaded Stud 114–20, 1.5 Long	8	1.09	8.72	McMaster Can orother Hardware Vendor	3l6StainlessSteel
90715Al25	114–20 Lock Nut (Nylock)	2	0.12	0.24	McMaster Can or other Hardware Vendor	316 Stainless Steel
90107A029	114 Flat Washer	14	0.07	0.98	McMaster Can or other Hardware Vendor	316 Stainless Steel

100,438 TOSSIT HYDROPHONE MOORING: Assembly drawing of the assembled TOSSIT mooring frame.

*100,440 FLOTATION RING: CAD* & PDF *file of one buoyancy floatation ring*.

100,441 LONG STANDOFF: CAD & PDF file of the long standoff post connecting the different sections of the mooring frame.

100,443 END PLATE: CAD & PDF drawing of the end-plate used at the top and bottom of the frame.

100,444 VEMCO MOUNT BUSHING: CAD & PDF drawing of the bushing used to attach the Vemco Ascent AR release to the lower end plate of the frame.

100,451 BATTERY HOUSING CLAMP: CAD & PDF drawing of the clamps used in the 4 section version of the frame to secure the battery housing into the frame.

100,452 SHORT STANDOFF: CAD & PDF file of the long standoff post connecting the different sections of the mooring frame.

100,453 ST300 HOUSING CLAMP: CAD & PDF drawing of the clamp used to secure the Soundtrap ST300 to the frame. This piece has to be adapted to use a different passive acoustic recorder in the TOSSIT mooring.

100,500 HANDLE: CAD & PDF file of the handle on top of the frame.

100,532 RELEASE LINK COLLAR: CAD & PDF file of release link collar used to remove spiel of the Vemco Ascent Release. This piece is attached to the release plug.

100,533 ANTI ROTATION COLLAR: CAD & PDF file of release link collar used to remove spiel of the Vemco Ascent release. This piece is attached to the release.

100,601 TOSSIT LINE PACK: CAD file of the line-pack assembly.

100,602 TOSSIT ANTI BACKLASH COLLAR: Assembly drawing of the Vemco Ascent AR release with the anti-backlash collar.

## Bill of Materials

**Build Instructions**

**Fig. 2:**
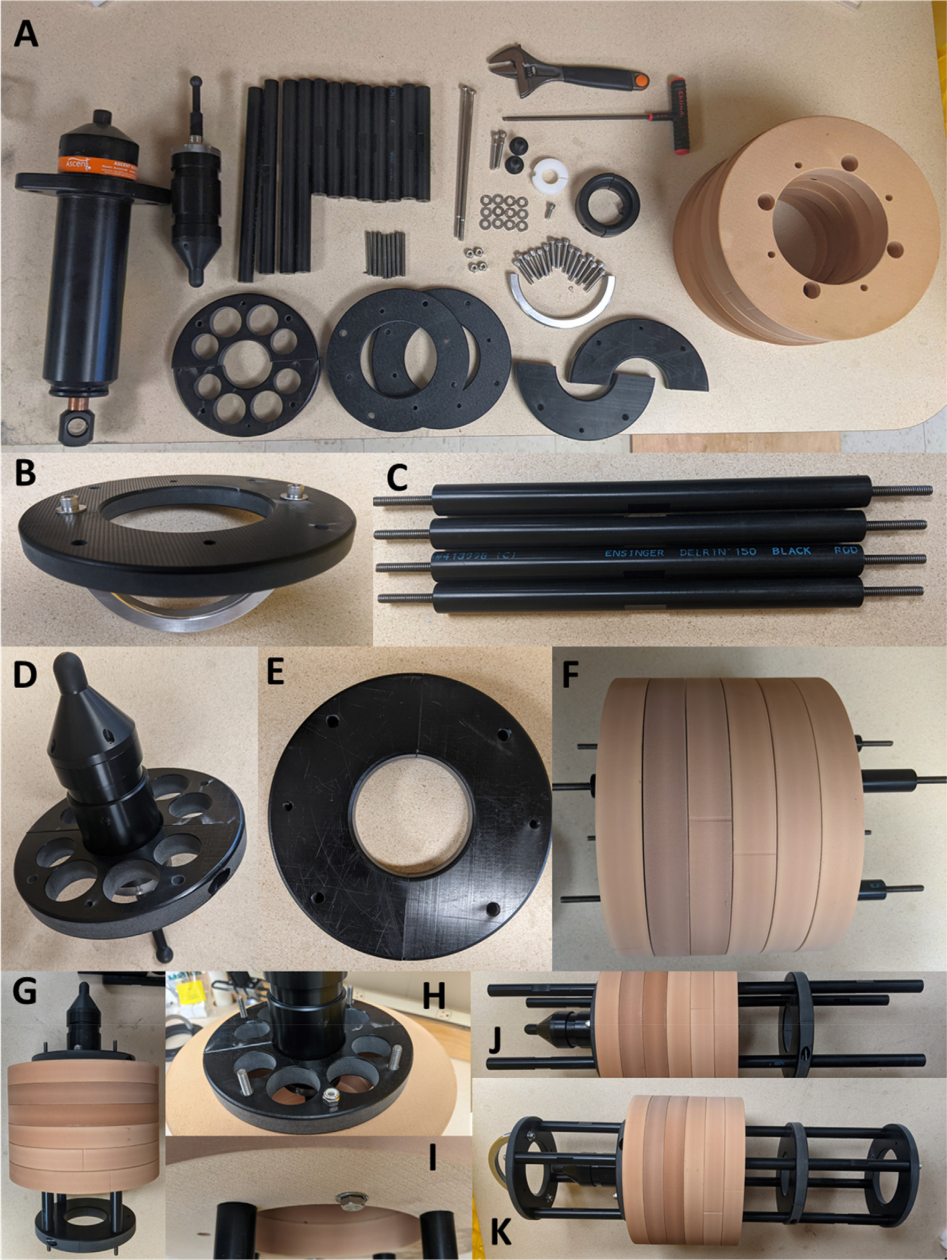
A) All parts needed for the assembly of a 3-section TOSSIT mooring frame. B – K, consecutive assembly steps.

**Fig. 3:**
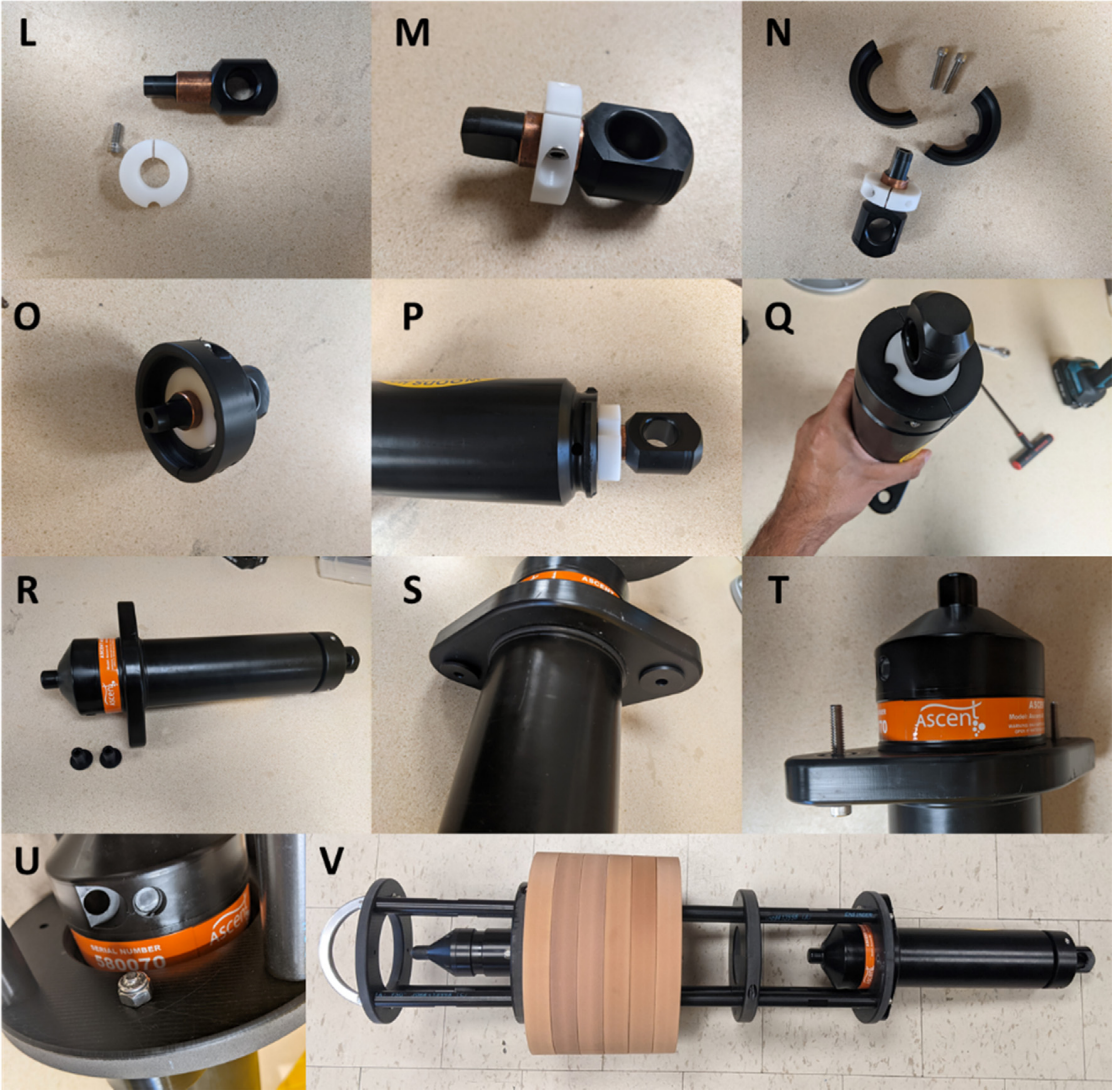
L-Q) Assembly steps of the anti-shock collar and ring. R-U) Steps to attached the acoustic release to the TOSSIT frame. V) Assembled TOSSIT mooring without anchor.

The TOSSIT hardware (screws, nut, bolts) can be acquired at your local hardware store. All Delrin parts and the buoyancy were produced at the machine shop at Woods Hole Oceanographic Institution, but can be produced at your local machine shop using the design files provided. An overview of all pieces required to assemble one 3 section TOSSIT is shown in Fig. 2A. For assembly, we provide a series of pictures Fig. 2B-K and Fig. 3L-V. For ease of assembly in the field where weight is relevant the TOSSIT frame can be constructed using a 3/16''  Allen key and an adjustable wrench. If available, a socket and impact driver speed up the construction significantly.

First we attach the handle (100500 HANDLE) to the top end plate (100443 END PLATE) with two ¼ inch 1.25 in. long machine head screws (92185A544) and washers (90107A029, Fig. 2B). Then we screw the studs (90575A546) into the long standoffs (100441 LONG STANDOFF), which form the basis for the buoyancy support (Fig. 2C). We chose this modular design so in case or a larger payload (e.g. longer acoustic recorder), the overall buoyancy could easily by increase by increasing the length of the long standoffs (100441 LONG STANDOFF) and adding more buoyancy plates. Pease note that the buoyancy fixation screw (92186A569) then needs to be chosen accordingly. In the next step, we assemble the Soundtrap fixture clamp (100453 ST300 HOUSING CLAMP) (Fig. 2D) which grips around the lower groove of the Soundtrap and is fixed with 2 screws (92185A544). If a different acoustic recorder is used, the Soundtrap holding plate is the piece that one would need to redesign.

Similarly, we put the two pieces together that form the battery housing clamp (100451 BATTERY HOUSING CLAMP, Fig. 2E) with 2 screws (92185A544).

In the next section we attach the floatation ring (100440 FLOTATION RING) to the frame. First, we align the floatation rings (6–8) and put the long standoffs through the respective holes (Fig. 2F) in the buoyancy plates. Then, we put the Soundtrap holding plate (Fig. 2D) on top and battery housing clamp (Fig. 2E) on the bottom of the buoyancy assembly (Fig. 2G). We fix the buoyancy with the long screws (92186A569) to the Soundtrap fixture clamp and secure it with a locking nut (90715A125, Fig. 2H&I). Then, we screw a short standoff (100452 SHORT STANDOFF) on each of the studs (Fig. 2J) and mount endplates (100443 END PLATE) using 4 screws (92185A544) and washers each (90107A029).

Now you should have finalized TOSSIT frame with the Soundtrap mounted. Assembly time is approximately 30 min.

After assembly of the TOSSIT frame, we equip the Vemco Ascent AR acoustic release with an anti-shock collar to remove all remaining movement between the lug and the release. First, we attach the release link collar (white, 100,532 RELEASE LINK COLLAR) with a ¼ inch machine head screw (92185A540) to the Vemco release lug (Fig. 3L&M). With the release link collar, we then fix the lug against rotational movement with the anti rotation collar (100533 ANTI ROTATION COLLAR, Fig. 3N&O). Fig. 3N&O are only for demonstration purposes. Before we mount the anti rotation collar, the lug with the release link collar needs to be attached to the Vemco release according to Vemco’s instructions. Once inserted, the anti rotation collar can be fitted into the Vemco’s release groove. The standard rubber O-ring that comes with the Vemco release is obsolete and can be removed. Please note not to overtighten the anti rotation collar. If overtightened, the friction between the anti rotation collar and the release link collar could prevent the release from working properly. Handtight is by far sufficient, and we advise to perform a function test of the release with the anti rotation collar and the release link collar mounted.

To attach the acoustic release to the lower endplate of the TOSSIT frame, two bushings (100444 VEMCO MOUNT BUSHING, Fig. 3R) are inserted into the acoustic release (Fig. 3S) and the release is attached from below with two ¼ inch × 2 in. machine head screws (92185A517) to the lower endplate (Fig. 3T), and fixed with self-locking nuts (90715A125, Fig. 3U).

Once the acoustic release has been attached to the frame, the TOSSIT is complete (Fig. 3V) and only needs to be attached to an anchor weight before deployment.

## Operation instructions

Operation of the TOSSIT mooring is straightforward. The Soundtrap and acoustic release have to be operated according to the manufacturers instructions and are not within the scope of this manuscript.

To deploy the assembled TOSSIT mooring only two steps are necessary: A) to attach the anchor weight to the mooring and B) to “toss it” overboard.

**Fig. 4:**
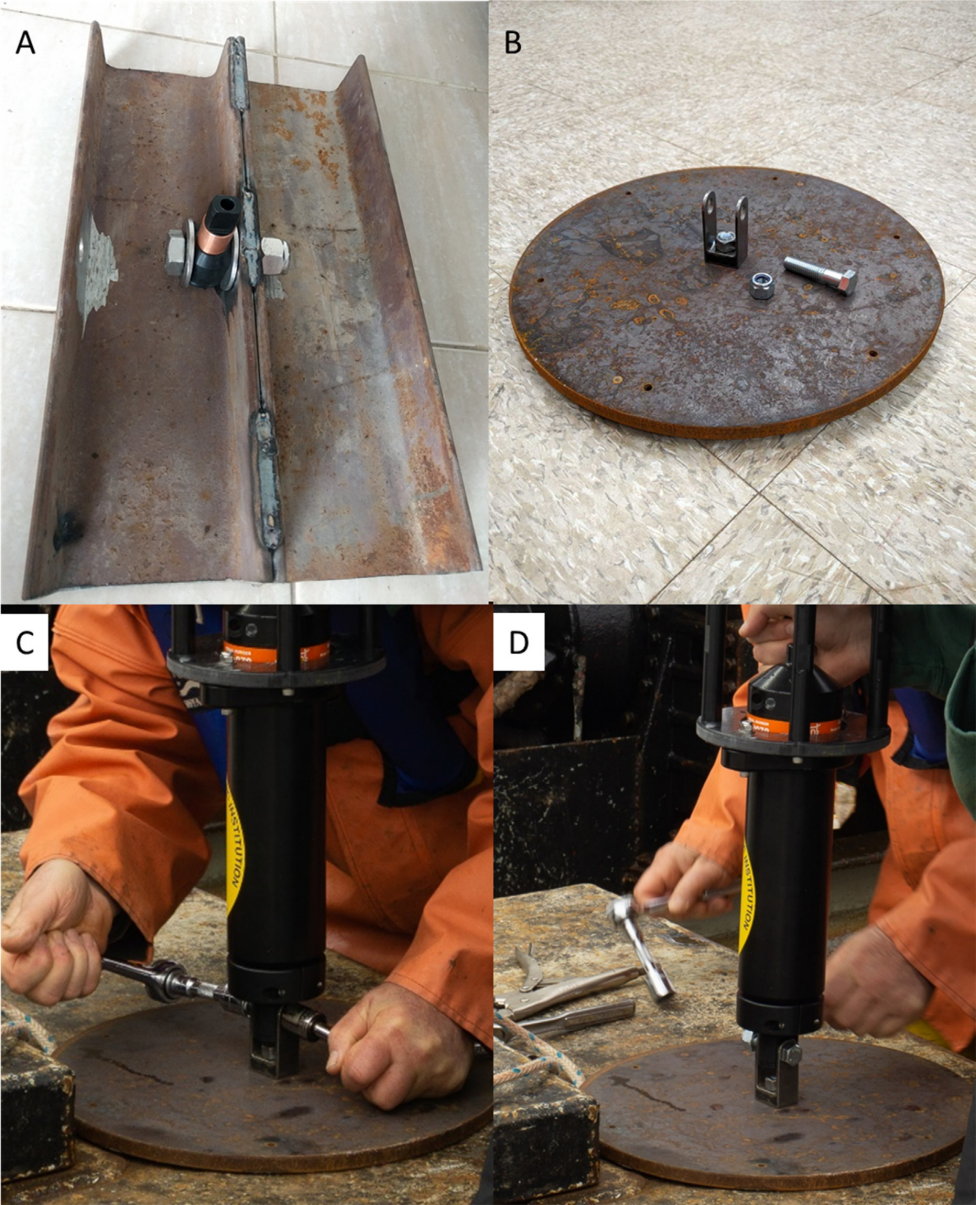
A) Anchor example created from two pieces of U-profile steel (∼15 kg) with attached mooring lug in the center. B) Anchor example created from a round steel plate (∼12 kg) with a bail to hold the mooring lug. C) Tighten the TOSSIT mooring lug using two nut drivers for fast attachment. D) Example of TOSSIT mooring attached to the anchor weight.

**Fig. 5:**
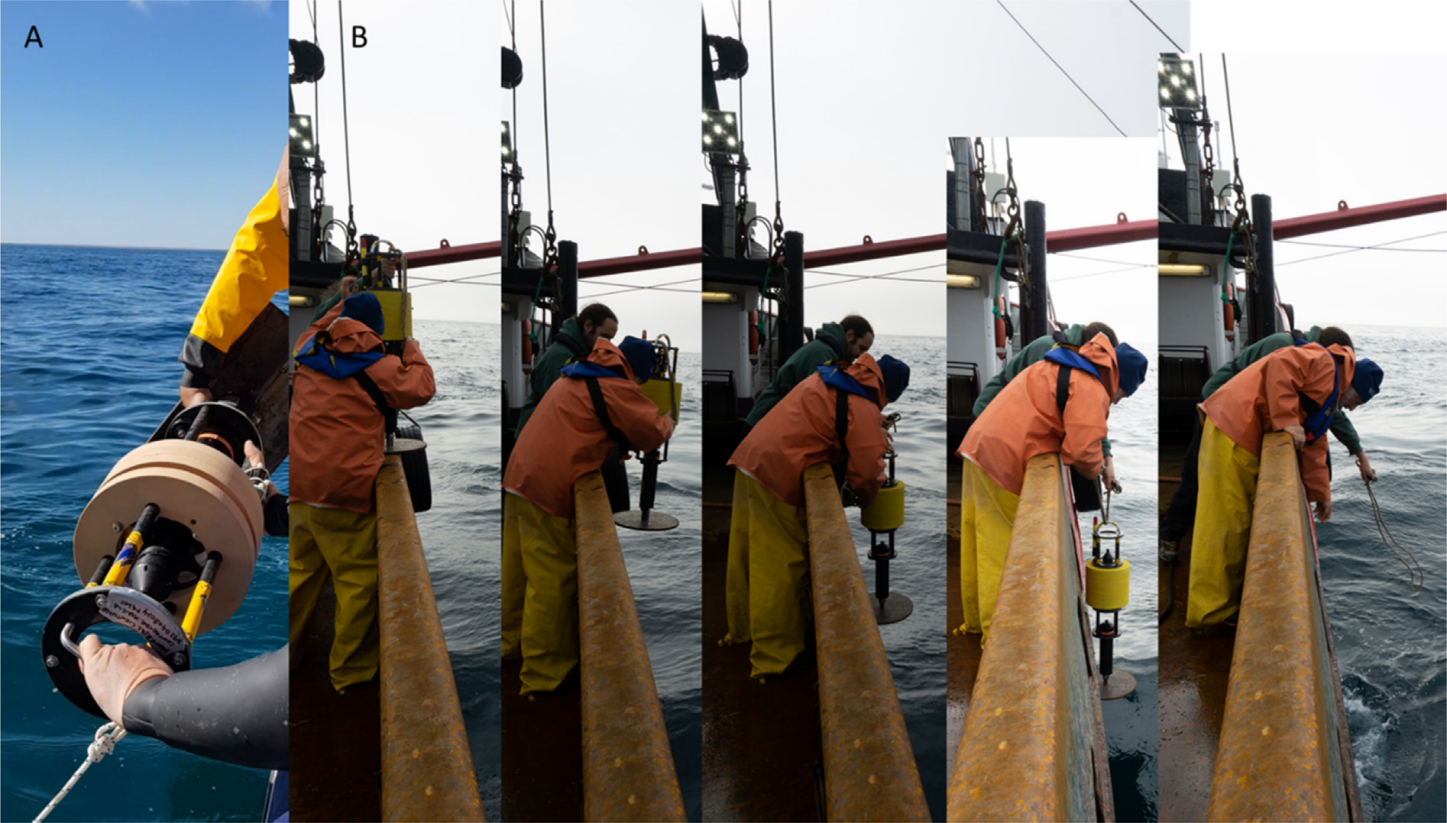
A) Horizontal deployment of a TOSSIT mooring from a small rigid hull inflatable boat (RHIB). B) Upright deployment of a TOSSIT mooring from F/V Kathryn Marie using a slip-line.

The anchor weight can have any form, it needs to provide a 1/2-inch hole where the acoustic release lug can be attached to. We used different anchor weight designs depending on what was available (Fig. 4A,B) at the deployment location. To mount the TOSSIT to the anchors, simply place the entire mooring system into the anchors mount, hold it steady and attach with and appropriate screw and locking nut (Fig. 4C). Ensure that the nut is tightened enough so the TOSSIT mooring cannot swivel. This is critical to provide low-noise data. The link between the mooring lug and the acoustic release is the weak link of the TOSSIT mooring. After the mooring is attached to the anchor weight and during release it is advisable to ensure that the mooring either stays upright (Fig. 4D) or is placed without any lever/weight on the mooring lug. We caution for the same during deployment. Ideally either hold onto the lug, lifting the weight when deploying the mooring horizontally (Fig. 5A). When deploying vertically from a vessel with a high water-line, a slip-line can be advantageous to avoid that the mooring might hit the vessel’s hull while tossing it over board (Fig. 5B). During retrieval use the handle to either lift the mooring by hand or boat-hook, depending on the vessel’s water-line.

## Validation and characterization

TOSSIT moorings have been used since 2018 for technical tests, marine mammal population studies as well as for environmental estimation experiments. We present Power Spectral Density data obtained during three experiments to validate the use of the TOSSIT mooring for low-cost passive acoustic monitoring. Advanced data analysis is out of scope of this article.

## San Antonio model Bay (SAMBAY) Experiment 2018

**Fig. 6:**
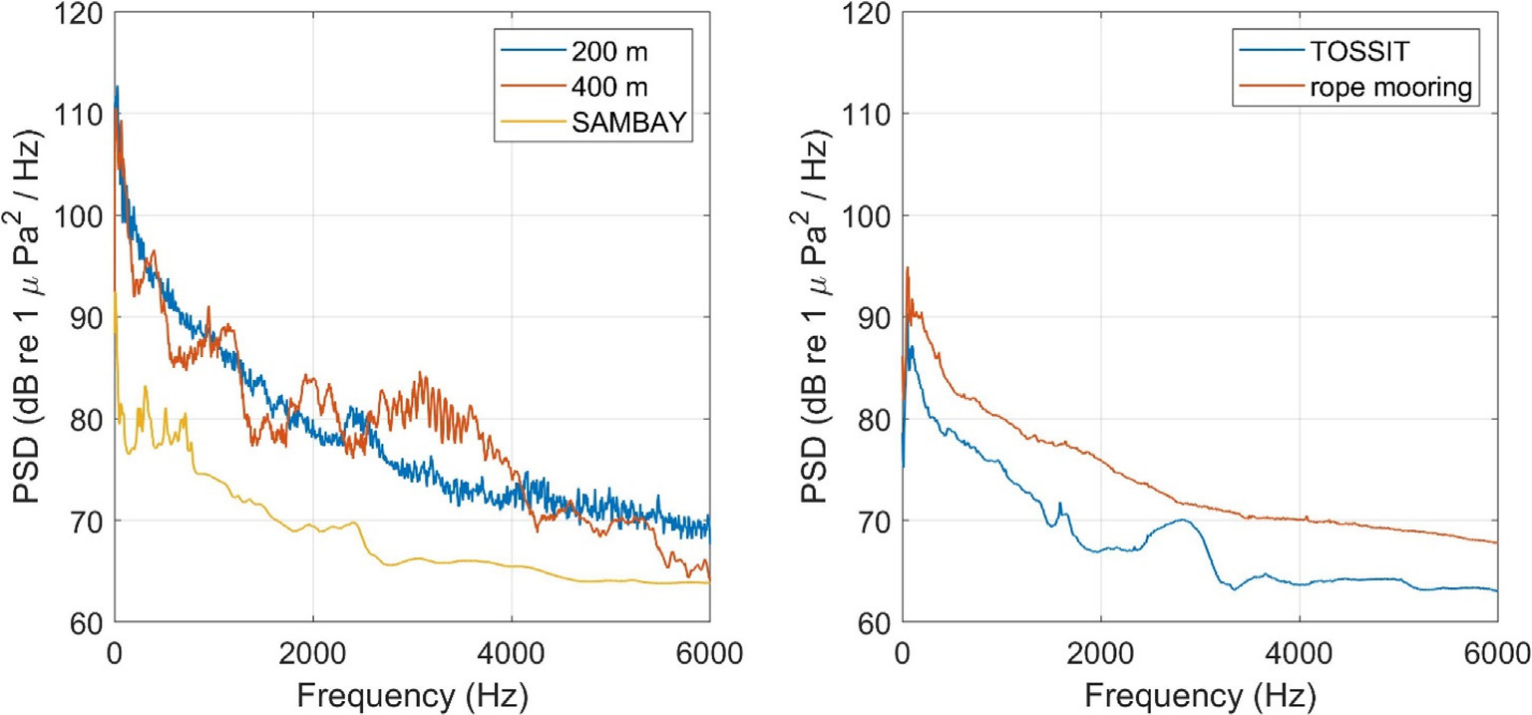
A) Power Spectral Densities (PSD) recorded using TOSSIT moorings at three different locations and water depths (SAMBAY 5 m, SBCEX 200 m, SBCEX 400 m). B) Power Spectral Densities (PSD) recorded a TOSSIT mooring and a classic rope mooring deployment in shallow water (16 m).

In the San Antonio Model Bay Experiment we aim to understand the influence of different covariates on the estimation of marine mammal density using passive acoustic monitoring. During the experiment, a passive acoustic monitoring distributed array is deployed in Baja San Antonio, Rio Negro, Argentina to record Southern Right whale calls (SRW) and SRW density data obtained from this PAM array is compared to concurrently collected visual and aerial SRW density data. In previous experiments (2015 and 2017) the PAM recorders were deployed and recovered by divers, in 2018 we made the first deployment of a TOSSIT mooring to test handling and feasibility. The TOSSIT mooring was deployed in 5 m water depth for 48 h on Sept 9th 2018 date by hand from a rigid hull inflatable boat. The anchor weight used was 15 kg steel u-profile welded together with a hole to accommodate the mooring lug (Fig. 4A). The 2018 TOSSIT version did not have an anti rotation collar on the acoustic release yet. The spectrum of the ambient noise is presented in Fig. 6A (blue curve). This example demonstrates the first operational use of the TOSSIT mooring, its capability to be deployed by hand from a small vessel (Fig. 5A) and successful recovery (Supplementary Video 1, https://doi.org/10.5281/zenodo.5632099).

## Buzzards Bay Experiment 2019

In 2019 we conducted a multi-week technical test of the TOSSIT mooring to compare it with traditional mooring methods. Two moorings were deployed of Stoney Beach, Buzzards Bay, MA in 16 m water depth on April 17, 2019 and recovered on May 7, 2019. One mooring consisted of a TOSSIT (with anchor recovery), the other mooring was connected the anchor to a surface buoywith a line and the Soundtrap was attached to the line. The spectrum of the ambient noise is presented in Fig. 6B. We find that the TOSSIT mooring shows lower ambient sound levels than the rope mooring which we attribute to the impact of the surface buoys movement due to waves and tide which can cause strumming of the line and broadband noise. This example shows that a Soundtrap recorder on a TOSSIT mooring provides recordings with lower self-noise levels, compared to a deployment with a mooring line and surface expression.

## Seabed characterization Experiment 2021

The Seabed Characterization Experiment (SBCEX) is an international, multidisciplinary, multi-institutional research project focusing on acoustic interaction and propagation in marine sediments. From 2015 to 2017, SBCEX focused on the ‘‘New England Mud Patch’’, a shallow water (depth ∼ 75 m) location about 95 km south of Martha’s Vineyard, MA, USA [8]. Several important SBCEX results were obtained using single receiver studies [18], [19], [20], [21], which motivated the use for TOSSIT for later experiments. In 2021, the SBCEX study area was extended to cover the deeper water of the New England Shelf Break, about 150 km south of Martha’s Vineyard with water depth up to 500 m. Six TOSSITs were used on the New England Shelf break in 2021 as part of SBCEX, and it is expected that up to 20 TOSSITs will be used in 2022 to cover both the Mud Patch and the Shelf Break. A subset of the 2021 TOSSIT data is presented here to demonstrate a relevant use case of the TOSSIT system.

A preliminary engineering trial was run in March 21, 2021. Of interest here is that a TOSSIT was successfully deployed and recovered at 39°57′35.00″N – 70°54′44.00″W, a location where water depth is 400 m. A spectrum of the local ambient noise is presented in Fig. 6A (yellow curve). The deployment was short (acoustic data collected on the seafloor from about 06 h40 to 11 h05 UTC), but it demonstrates the TOSSIT capacity to operate near its theoretical depth limit (500 m).

A set of six TOSSITs were later deployed as part of the main 2021 SBCEX, along with many other acoustic assets including receivers (vertical line arrays, vector sensors) and acoustic sources. The TOSSITs were deployed and recovered in various locations with water depth between 200 and 300 m. Acoustic data was successfully collected on all the TOSSIT during their whole deployment, from May 22 to June 02. As an example, we focus here on a TOSSIT deployed at 40° 2′60.00″N – 70°51′35.00″W. The spectrum of the ambient noise is presented in Fig. 6A (brown curve). This example demonstrates the capacity of TOSSIT to perform longer deployments (∼2 weeks), and to be used as part of large-scale ocean acoustic experiments.

The 2 TOSSIT deployments presented in this section were performed from the F/V Kathryn Marie, a 74′ long scallop fishing vessel. Preliminary trials (not presented here) were also performed (September 2020) on the New England Mud Patch from the FV Gunsmoke, a 14′ long sport fishing vessel. This further demonstrates the possibility to use TOSSITs from various platforms of opportunity.

**Funding**.

*The development of the TOSSIT mooring was supported by a Woods Hole Oceanographic institution Innovative Technology Award (Award number 25226). TOSSIT deployment in Argentina was supported by a Woods Hole Oceanographic Institution Mary Sears visitor award (Award number 24700) and TOSSIT deployments during SBCEX were funded by the Office of Naval Research Task Force Ocean (ONR TFO, Award number: N000141912627). The funders had no role in study design, data collection and analysis, decision to publish, or preparation of the manuscript.*

**Human and animal rights**.

If the work involves the use of human subjects, the author should ensure that the work described has been carried out in accordance with the appropriate ethical guidelines.

No human or animals involved in the study.

## Declaration of Competing Interest

The authors declare that they have no known competing financial interests or personal relationships that could have appeared to influence the work reported in this paper.
